# Surface Modification of Aliphatic Polyester to Enhance Biocompatibility

**DOI:** 10.3389/fbioe.2019.00098

**Published:** 2019-05-03

**Authors:** Yazhong Bu, Junxuan Ma, Jianzhong Bei, Shenguo Wang

**Affiliations:** ^1^Beijing National Laboratory for Molecular Sciences, State Key Laboratory of Polymer Physics and Chemistry, Institute of Chemistry, Chinese Academy of Sciences, Beijing, China; ^2^Orthopedic Research Institute, The First Affiliated Hospital of Sun Yat-sen University, Guangzhou, China; ^3^Guangdong Provincial Key Laboratory of Orthopedics and Traumatology, Guangzhou, China

**Keywords:** biomedical material, aliphatic polyester, biocompatibility, surface modification, tissue engineering

## Abstract

Aliphatic polyester is a kind of biodegradable implantable polymers, which shows promise as scaffolds in tissue engineering, drug carrier, medical device, and so on. To further improve its biocompatibility and cell affinity, many techniques have been used to modify the surface of the polyester. In the present paper, the key factors of influencing biocompatibility of aliphatic polyester were illuminated, and the different surface modification methods such as physical, chemical, and plasma processing methods were also demonstrated. The advantages and disadvantages of each method were also discussed with the hope that this review can serve as a resource for selection of surface modification of aliphatic products.

## Introduction

Biodegradable polymers are defined as materials whose chemical and physical characteristics undergo deterioration and complete degradation when exposed to certain conditions, which have many important applications in medical and related fields (Rezwan et al., 2006; Nair and Laurencin, 2007). These polymers can be divided into natural biodegradable polymers and synthetic biodegradable polymers. Natural materials mainly include polysaccharides and proteins. Although they have good biocompatibility and some biofunctions, their strong immunogenic response, complex purification process and disease transmission possibility limit their applications. The synthetic biodegradable polymers can be prepared with designed routes, so they have more predictable properties and batch-to-batch uniformity. There are many synthetic biodegradable polymers, such as aliphatic polyesters, polypropylene fumarate, polyhydroxyalkanoates, and so on. Among them, aliphatic polyesters, including polyglycolide (PGA), polylactide (PLA), polycaprolactone (PCL), and their copolymers (copolylactones), are the most often used ones in tissue engineering and other bio-medical applications. Aliphatic polyesters have many advantages. Under the physiological environment, polymeric chains of the aliphatic polyesters can fracture into small pieces, with the molecular weight of the pieces decreasing from high to low. The polymeric pieces become dissolvable and finally will be absorbed or metabolized *in vivo*. In this way, not only the formation of foreign body reaction can be avoided, but also the secondary surgery for removing the foreign matter can also be avoided. Compared with the natural biodegradable polymers, aliphatic polyesters have the adjustable degradation rate, excellent processability, high mechanical strength, easiness to sterilization as well as good reproducibility and low price (Drury and Mooney, 2003). Therefore, aliphatic polyesters have become a kind of important biomedical polymer materials and have been approved as an implantable biomaterial for using *in vivo* by Food and Drug Administration (FDA) of many countries including China, USA and European.

## The Influencing Factors of Biocompatibility of Aliphatic Polyester

Indeed, biocompatibility includes the interactive mechanisms relating the biomaterial with its biological environment (Lotfi et al., 2013). Since the materials interact with tissues through the cell adhesion, the biocompatibility of materials is very closely related to cell adhesion (Lotfi et al., 2013). Generally, the response of cells to a surface is dictated by the interactions of the proteins and the substrate (Kasemo and Lausmaa, 1994; Anselme, 2000; Oliveira et al., 2011). Firstly, proteins adsorb onto the surface of the material, being followed by the binding of cellular membrane receptors to the chemical groups of those proteins or even directly to the substrate exposed chemical groups. The proteins adsorption is often caused by physical (Van Der Waals force) or chemical action (ionic interaction), which happens very quickly. Then, the cell attachment is caused by the interaction between cells and some bioactive molecules, such as extracellular matrix protein, membrane proteins, cytoskeleton proteins, and so on. By controlling the cell adhesion on materials, the biocompatibility of materials can be optimized (Anselme, 2000; Veiseh et al., 2015; Chaudhuri et al., 2016). Generally speaking, the aliphatic polyester is biocompatible (Rasal et al., 2010). It will not produce toxic or carcinogenic effects in local tissues. Also the degradation products will not interfere with tissue healing (Athanasiou et al., 1996). However, for some applications, the biocompatibility is still need to be improved, because polyester intrinsically is too hydrophobic and lack reactive side-chain groups (Liu et al., 2018). Given that the surface of biomaterials will contact the cells before all other parts, the properties of the surface are particularly important for the cell adhesion and later the biocompatibility and these properties are summarized as follows.

### Wettability

Wettability, also known as the hydrophilicity and hydrophobicity, will influence the cell attachment and protein absorption. The cell adhesion is induced by the protein and mediated by receptors on the cell membrane. In the cell adhesion process, the proteins are firstly absorbed onto the surface of the material, after which cellular membrane receptors are bound to proteins' chemical groups or directly to the chemical groups of the substrate (Kasemo and Lausmaa, 1994). Generally, hydrophobic surfaces will display a better affinity for proteins (Lampin et al., 1997; Yousefi et al., 2018). Therefore, the surface of the material must have hydrophobicity to absorb protein to help the recognition (Hynes, 1992). However, the hydrophilic surfaces possess a higher affinity toward the cells. A number of studies have shown that enhancing the hydrophilic properties of polymers leads to increased cell spreading and adhesion (Lee and Lee, 1993; Allen et al., 2006). As a result, keeping a balance between hydrophobicity and hydrophilicity is vital to ensure the protein adsorption as well as the cell growth (Good et al., 1998; Wang Y. W. et al., 2003).

### Electric Properties

The cell membrane is negative (Wen et al., 2016). So generally positive materials are good for cell adhesion and negative surface will have charge repulsion with cells (Steele et al., 1995; Hoshiba et al., 2018). However, there are reports about a newborn rat calvaria bone osteoblasts adhering in both positive and negative surface of the polystyrene ion exchange resin microspheres (Gao et al., 1998), in which protein adsorption and cell migration can be greatly enhanced. Still the data showed that different kinds of charged surfaces led to obviously different cell morphology. So, the electric properties of the materials, including the types and densities, have great influence on the cell adhesion behavior (Shirazi et al., 2016; Kuo and Rajesh, 2017).

### Surface Free Energy

Surface free energy can affect polar molecules such as water and protein and high surface free energy of the material is more advantageous to cell adhesion and spreading (Vandervalk et al., 1983; Nakamura et al., 2016; Ueno et al., 2019). Nakamura et al. reported that changes in surface free energy would affect polar molecules such as water and proteins. The increase in surface free energy will improve the wettability and then accelerate the cell adhesion (Nakamura et al., 2016). The surface free energy depends on chemical composition, functional groups and electric properties of material surface. These surface properties are mainly decided by the physical and chemical properties of the materials (Vanpelt et al., 1985; Nakamura et al., 2016).

### Surface Morphology

#### Roughness

**Figure 1:**
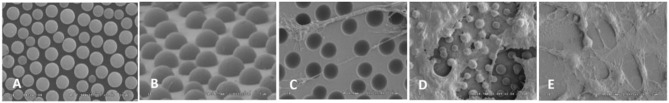
**(A)** PS surface with micrometer scale pits, × 10,000; **(B)** PLLA surface with micrometer scale islands, × 10,000; OCT-1 osteoblasts on **(C)** PS surface with micrometer scale pits (× 5,000), **(D)** PLLA surface with micrometer-scale islands (× 2,500), and **(E)** smooth PLLA surface (× 1,200) (Wan et al., 2005) [Readapted with permission from Wan et al. (2005). Copyright 2004 Elsevier Ltd.].

**Figure 2:**
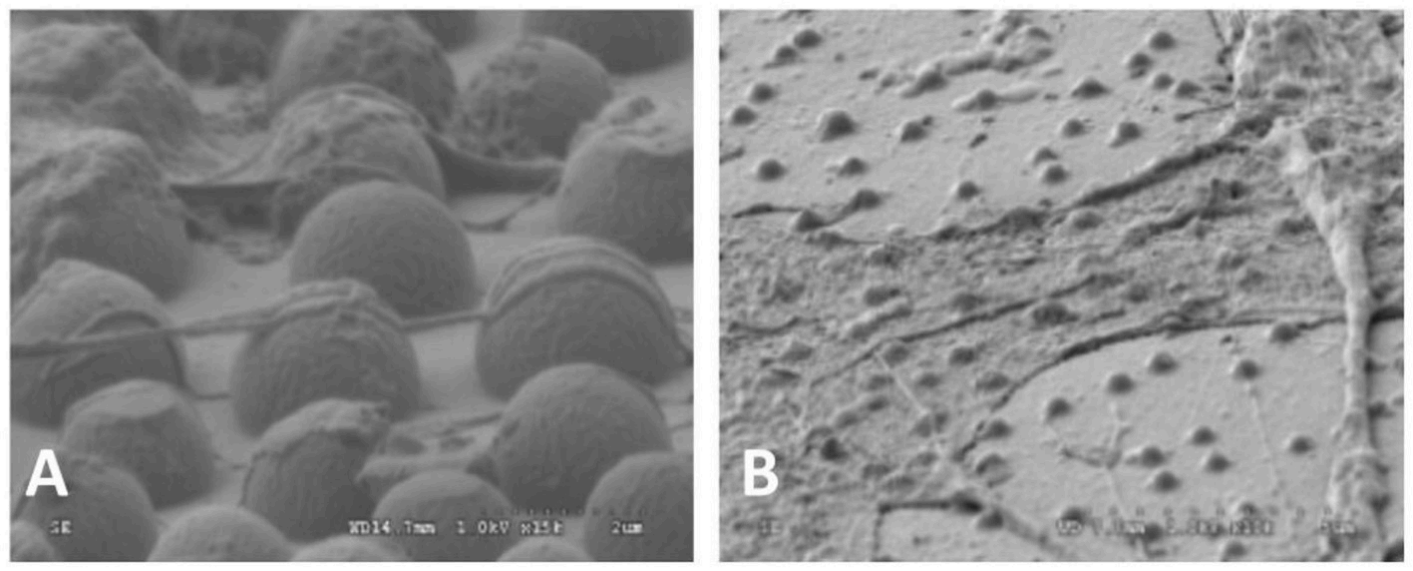
SEM images of OCT-1 osteoblasts on PLLA surface with micro-island for 6 h: **(A)** the surface with micrometer scale islands, × 25,000; **(B)** The surface with nanometer scale islands, × 10,000 (Wan et al., 2005) [Readapted with permission from Wan et al. (2005). Copyright 2004 Elsevier Ltd.].

Rough surface can enhance the cell adhesion by influencing the protein adsorption and providing larger area for cell adhesion compared with smooth surface (Lampin et al., 1997). Rough surface is good for the formation and grow of biofilm (Quirynen and Bollen, 1995). Wan et al. found that although OCT-1 osteoblast could adhere to smooth, pits-patterned, and island-patterned surface of PLLA (Figures 1A,B), they had differences in their state of spreading. On the pits- and island-patterned surfaces (Figures 1C,D), the cells spread better than those on the smooth surface (Figure 1E). The height of cells on pit-patterned surface was obviously lower than that on the flat ones (Wan et al., 2005). It was observed that cells could adhere onto the islands and grow along the convex surface of the islands. It was also observed that the tiny pseudopodium protrusions strode from one island to another as shown in Figure 2. Because cells had more contact area and spread better on the rough surface than on the smooth ones, the OCT-1 cells had lower height on rough surfaces as was shown in Figure 1.

**Figure 3:**
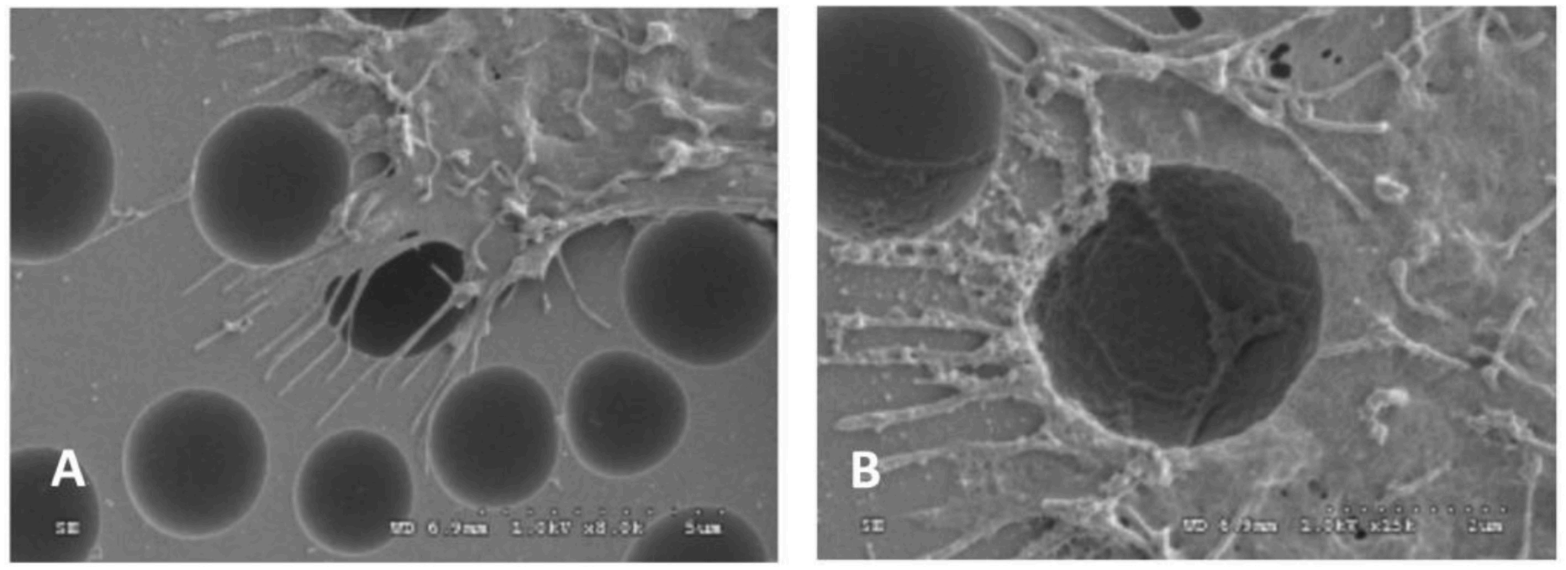
SEM images of OCT-1 osteoblasts on PS surface with micro-pits for 6 h: **(A)** for micrometer scale pits (2.2 μm), × 8,000; **(B)** for micrometer scale pits (2.2 μm), × 15,000 (Wan et al., 2005) [Readapted with permission from Wan et al. (2005) Copyright 2004 Elsevier Ltd.].

It could be seen that the cells could stride over the pits with 2.2 μm of radius (Figure 3A). The pseudopodiums of the cells could intrude inside the pits and grow along the curvature wall of the pits because the pseudopodiums of the cells had less rigidity compared to the bulk of the cells, showing “contact guidance” effects (Figure 3B). These pseudopodiums acted as anchoring points to pull the cell body, suggesting that cell is allowed to penetrate and proliferate on scaffolds with this pore size. Filopodia of cells would alter their original orientation to grow along the ridge of the pits when they reached the borders, which might be caused by “groove-ridge” induced “contact guidance”. However, according to Wan, cells could not grow inside nano-scaled pits with the diameter to be 0.45 μm (Curtis and Wilkinson, 1997; Wan et al., 2005).

**Figure 4:**
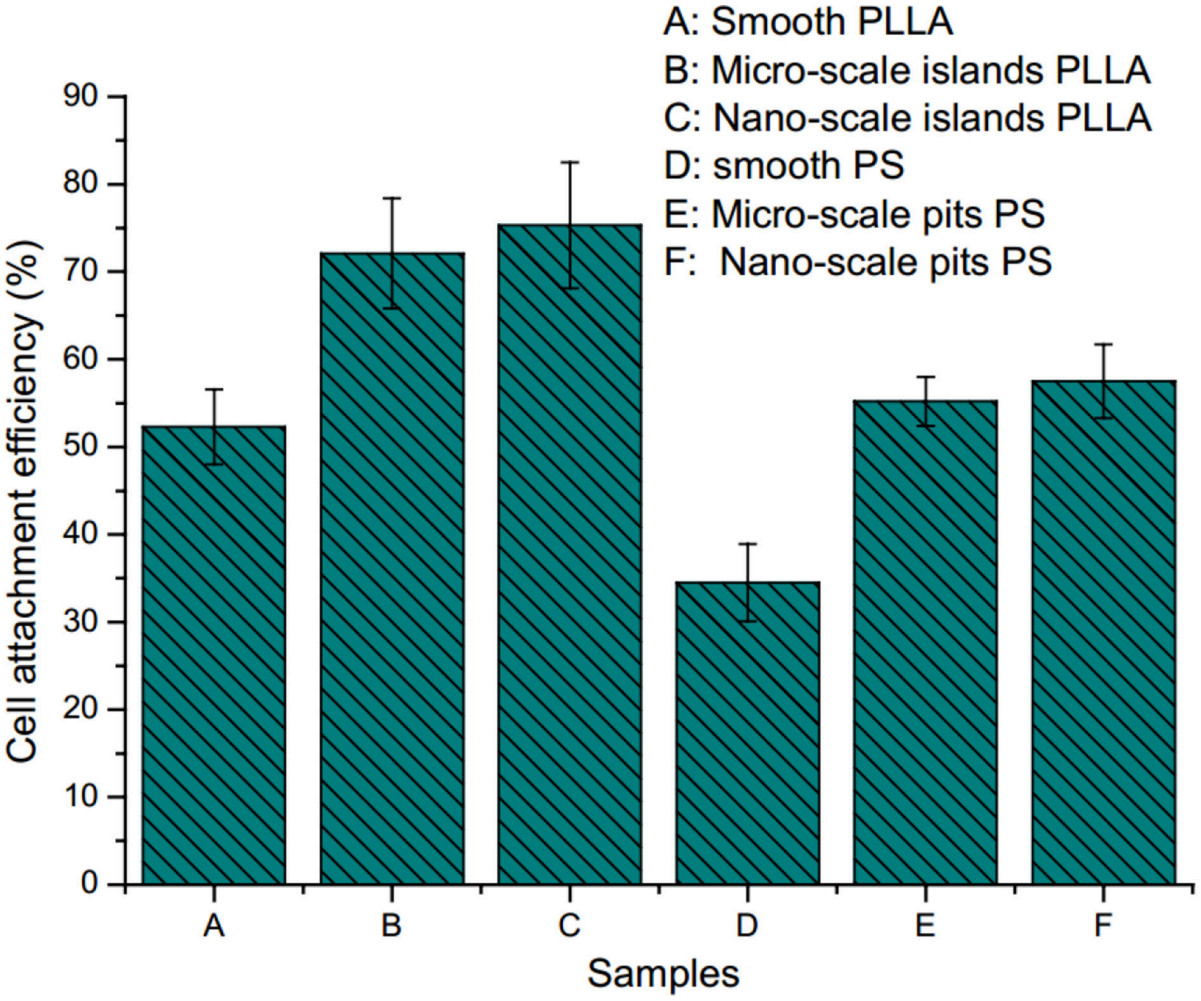
Cell attachment efficiency on polystyrene and poly(l-lactide) surface with different sized pits and islands: **(A)** on smooth PLLA surface; **(B)** on PLLA surface with micro-scale islands; **(C)** on PLLA surface with nano-scale islands; **(D)** on smooth PS surface; **(E)** on PS surface with micro-scale pits; **(F)** on PS surface with nano-scale pits (Wan et al., 2005) [Readapted with permission from Wan et al. (2005). Copyright 2004 Elsevier Ltd.].

It also found that surface morphology not only affected the cell adhesion and growth, but also the adhesion efficiency. Figure 4 demonstrated that, only around 30% of the osteoblasts could adhere on the smooth surface, while much more could adhere on rough surface (50–75%).

However, generally speaking, the response of cells to roughness is different depending on the cell type (Chang and Wang, 2011). For osteoblasts and neurons, which is large, they might need larger surface roughness (Donoso et al., 2007). For smaller cells, like human vein endothelial cells, nano-scale of roughness could enhance cell adhesion and growth (Chung et al., 2003).

#### Microgrooves

**Figure 5:**
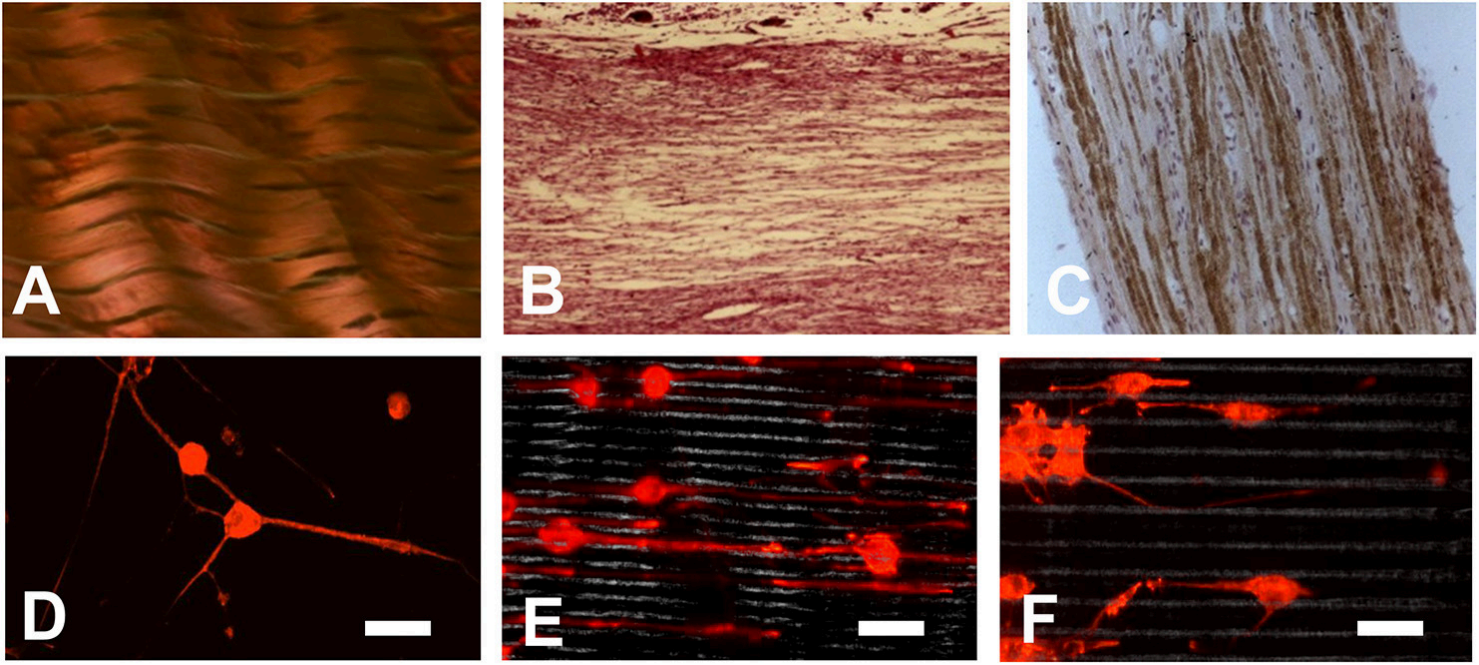
**(A–C)** Some natural tissues have a parallel orientation structure: **(A)** Tendon; **(B)** Peripheral nerve; **(C)** Spinal cord; **(D–F)** Orientation of neurite growth on laminin peptide coated PLGA scaffold after 6 days culture **(D)** on smooth surface; **(E)** on scaffold with microgrooves of 5 μm; **(F)** on scaffold with microgrooves of 10 μm (Yao et al., 2009). (Bar scale, 60 μm) [Readapted with permission from Ref. (Yao et al., 2009). Copyright 2008 Acta Materialia Inc. Published by Elsevier Ltd.].

Some natural tissues have the parallel orientation structures such as tendon, peripheral nerve and spinal cord (Figures 5A–C), so the scaffolds for tissue engineering will be more promising if they can stimulate the parallel orientation structure. It was proved that by using substratum with certain shapes, alignment, or directional growth of cells in the developing brain could be induced (Hatten, 1990). By using laser ablation methods, Yao et al. fabricated micropatterned PLGA films. After being coated with collagen type I or laminin peptide (PPFLMLLKGSTR), these films showed a guidance effect on both early stage neurite outgrowth and elongation (Figures 5D–F) (Campbell and von Recum, 1989; Yao et al., 2009).

### Surface Structure

Surface structures, such as walls, edges, or holes, influences the motility and spreading of cells and can be used to control the direction and localization of cell growth. Porous structure were reported to be conducive to the nutrients penetrate and cell metabolism, which was good for cell adhesion and growth (Kuo et al., 1997). Richter et al. reported that nylon net with smaller pores had larger specific surface area than those with larger pores and more cells grew on surface of materials with smaller pores. This was because cells could penetrate the nylon net with large pores, leading the failure of cell adhesion on the surface (Richter et al., 1996). So it is preferred that the scaffold for tissue engineering have porous structure and the cell adhesion behavior could be tuned by the size of the pores (Cai et al., 2002).

In summary, factors influencing the cellular affinity and then biocompatibility are summarized in Table 1. To improve the cellular affinity and biocompatibility, the modification must be used according to practical application purpose and the physical-chemical properties.

**Table 1:** The factors and influences of the material surface on biocompatibility and cell affinity.

**Factors**	**Influences**
Hydrophilicity/hydrophobicity (Yousefi et al., 2018)	Proper hydrophilicity is beneficial to cell adhesion and growth
Surface free energy (Nakamura et al., 2016)	High surface free energy is beneficial to cell adhesion and spread
Surface electricity (Koo et al., 2018)	Positive electricity is beneficial for attracting cells
Surface structure (Bacakova et al., 2011)	Roughness surface is beneficial for cell adhesion and biological membrane growth

## Surface Modification of Aliphatic Polyester

Aiming at the above surface properties for biocompatibility, there are many ways to modify aliphatic polyester to improve the surface properties and the biocompatibility (Liu et al., 2018; Miele et al., 2018). The commonly used methods for modification are summarized in Table 2.

**Table 2:** The surface modification methods for polylactone-type products.

**Categories**	**Ways**	**Mechanism**
Chemical modification	Bulk modification (Cui et al., 2005; Phelps et al., 2012)	Copolymerization of various monomers
	Surface grafting (Lih et al., 2008)	Adding various functional groups to the surface
Physical modification	Surface coating (Yang et al., 2018)	Covering the surface with biocompatible materials
Plasma modification	Low temperature plasma treatment (Wang et al., 2004)	Changing the topological structure of surface
	Plasma treatment-biomolecule anchoring method (Shen et al., 2009, 2010)	Anchoring bioactive molecules on the surface by using the charged groups on the surface

### Bulk Modification—Copolymerization

By means of bulk modification, which is to copolymerize hydroxyl acid monomer with the molecules containing hydrophilic or charging groups (carboxyl group, hydroxyl group, amine group, subamine group, sulfonic group, amide group, etc.), surface properties of the polymers, such as crystallinity, hydrophilicity, the types and quantity of charging, and reactive groups, can be changed and optimized, finally enhancing the cell adhesion and cell affinity (Wang, 2002; He et al., 2004b; Cui et al., 2005; Wang S. G. et al., 2005; Shenguo and Jianzhong, 2011; Qiang et al., 2014; Amani et al., 2019). Many monomers and polymers can be used for the copolymerization. Poly(ethylene glycol) (PEG) is a highly biocompatible, nontoxic material with excellent hydrophilicity (Phelps et al., 2012; Rong et al., 2019). To enhance hydrophilicity of the poly(L-lactic acid) (PLLA), Chen et al. used poly(ethylene glycol) (PEG) macromolecular monomer to copolymerize with PLLA. With higher content of the ethylene glycol (EG) unit, water uptake ability of the PLLA-co-PEG copolymer (PLE) increased (Chen et al., 2002).

**Figure 6:**
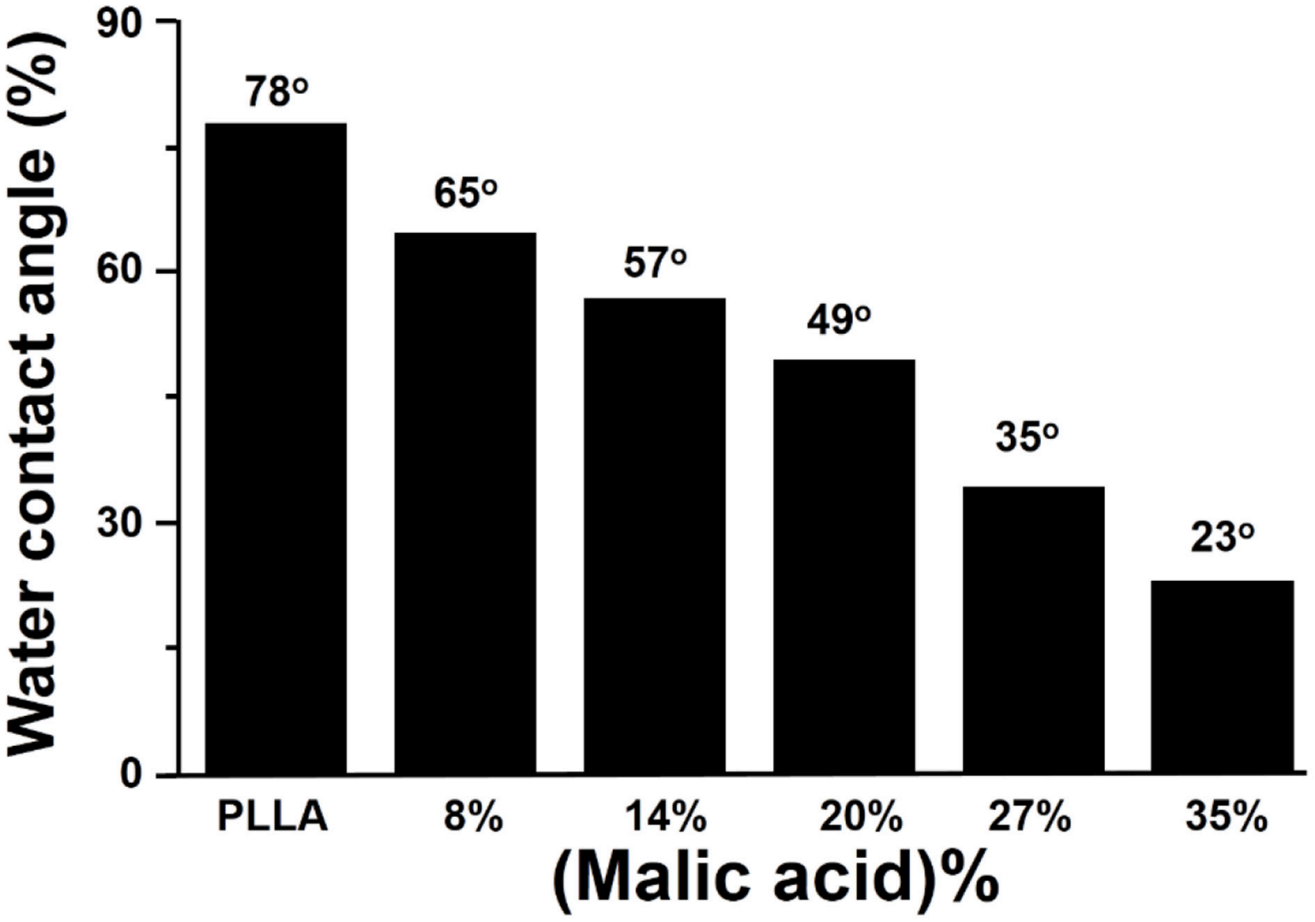
Effect of malic acid content on water contact angle of PLMA (He et al., 2004b) [Readapted with permission from He et al. (2004b). Copyright 2003 Elsevier Ltd.].

**Figure 7:**
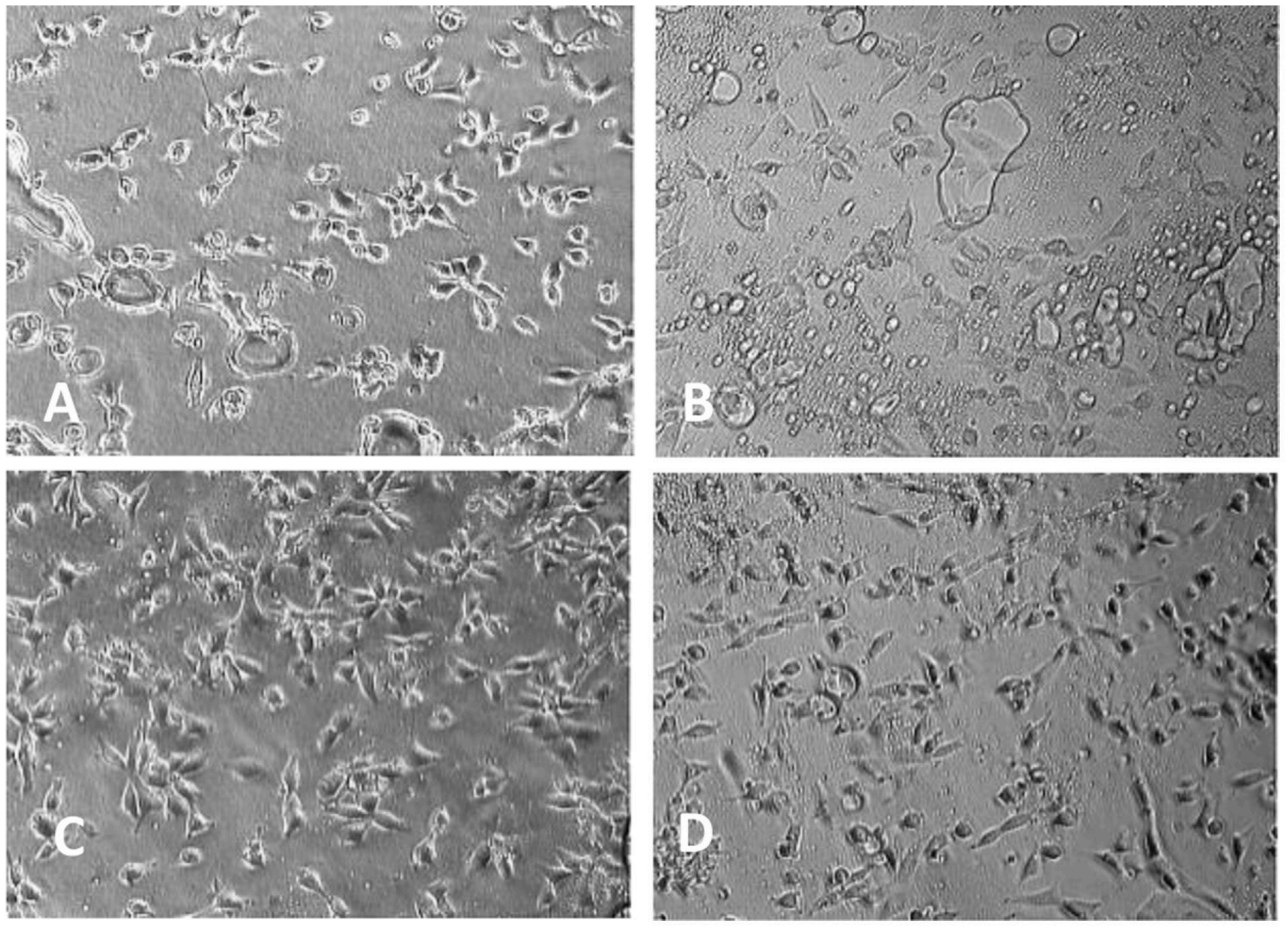
Morphology of 3T3 mice fibroblasts cultivated on PLMA with different composition for 5 h (× 150): **(A)** PLLA; **(B)** PLMA (96/4); **(C)** PLMA (92/8); **(D)** PLMA (86/14) (He et al., 2004b) [Readapted with permission from He et al. (2004b) Copyright 2003 Elsevier Ltd.].

Aliphatic polyester lacks recognition sites for cells. By copolymerization of lactone monomer with other monomers containing pendant carboxyl groups and/or amino groups, it would be more easily for bioactive agents to immobilize on the material surface, enhancing the cell adhesion. He et al. used lactide to copolymerize malic acid (MA) with two carboxyl groups, and the hydrophilicity of resulting copolylactone (PLMA) could be tuned by altering the ratio of MA. With the increased amount of MA, the contact angles of the PLMA decreased (Figure 6) (He et al., 2003, 2004a,b). By comparing the rat 3T3 fibroblasts on the surface of PLMA with different amount of MA after 5 hours, the cells showed different morphology on the surface. PLMA (96/4) and PLMA (92/8) showed better attach efficacy than pure PLLA and PLMA (86/14) (Figure 7) (He et al., 2004b). One of the degradation products of PLMA, MA is a natural component of juice and also a necessary organic acid for human beings. So just like LA, MA could be metabolized and absorbed by bodies, which would not cause any side effects.

Because that the natural polymers are usually biodegradable and offer excellent biocompatibility, as well as good cell affinity, they could also be used to copolymerize with aliphatic polyesters. For example, by using the trimethylsilyl-protected (TMS) dextran as macroinitiator, Cai et al. synthesized PLA grafting dextran (PLA-g-dextran) and proved that compared PLA-g-dextran with the pure PLA, the PLA-g-dextran copolymer exhibited not only better hydrophilicity but also better cell affinity (Cai et al., 2003). Qu et al. also reported that after grafting lactic acid onto amino groups of chitosan, a novel pH-sensitive physically crosslinked hydrogels could be constructed (Qu et al., 1999).

### Surface Grafting

After polymerization of glycolide, lactide, and caprolactone, there are still some functional groups at end of the polylactone chains. By using these functional groups, the hydrophilic groups and/or charged groups can be grafted onto surface of the aliphatic polyesters through chemical reaction to improve cell compatibility, blood compatibility as well as anticoagulation properties. Since the chemical bonding is strong and stable, a long-term modification effect can be realized.

Heparin is a natural anticoagulant substance. It is used to inhibit prothrombin activation, slow down and stop formation of the fibrin network. It can also prevent incidence of infection. Heparin has been also used to improve the anticoagulant properties of polyester. By using Michael-type addition between thiolated heparin and PLGA-PEG-PLGA diacrylate, Lih et al. developed novel heparin–conjugated polyester hydrogels. This hydrogel exhibited temperature dependent sol-gel transition behavior and might be used as injectable scaffold (Lih et al., 2008). Except for covalently immobilizing heparin on PLGA surface, Wang et al. also graft chitosan on the surface of PLGA using N-(3-dimethylaminopropyl)-N-ethylcarbodiimide (EDC) and N-hydroxysuccinimide (NHS). After grafting, the water contact angle of the modified film was greatly decreased and the blood and cell compatibility was improved (Wang X. H. et al., 2003). Compared with the surface coating method, surface grafting method will lead to tougher bonding between biofunctional molecules and the surface, which is expected to play a more and more important role in the field of biomedical applications. However, it is worth noting that the aliphatic polyester is lack of functional groups, of which only the end of main chains have the functional groups. The ones exposed on surface of the aliphatic polyester are even less. Even if all these functional groups are modified, the surface modification effect is still limited. On the other hand, sometimes organic solvents might be used in the chemical modification, which might cause destruction of topology structure of the surface as well as pollution of the environment.

### Surface Coating

Because the different solubility of natural polymers and the synthetic polyesters, in most cases, bulk modification was carried out using synthetic polymers, which lacked biomedical functions because of the intrinsic shortcomings of synthetic polymers. In order to add some biofunctions to the polyesters, many natural biofunctional materials such as hyaluronic acid, protein, lipid, collagen, polysaccharide, peptides, and gelatin, are coated on the surface of synthetic polymers. Generally, the coating materials are prepared in solutions and the coatings were prepared by soaking, brushing, or spraying methods. Hyaluronic acid and collagen possess excellent biocompatibility and cell affinity which are the mostly used coating materials for surface modification (Yang et al., 2018). However, although surface coating method is simple and effective, this method is still a physical treatment and the coating has bonded to the surface by Van Der Waals force, which is relatively weak. Especially in the presence of water or body fluids, the coatings are easily to dissolve and break away from the surface, shortening the duration of the surface treatment (Balaji et al., 2015). Besides, some solvents may destruct the topology of polyester, causing adverse effects on cell affinity. Some coating fluids are too viscous, which might change the topology of the original surface, and could not infiltrate into materials due to their high viscosity.

### Plasma Modification

#### Plasma Modification

**Figure 8:**
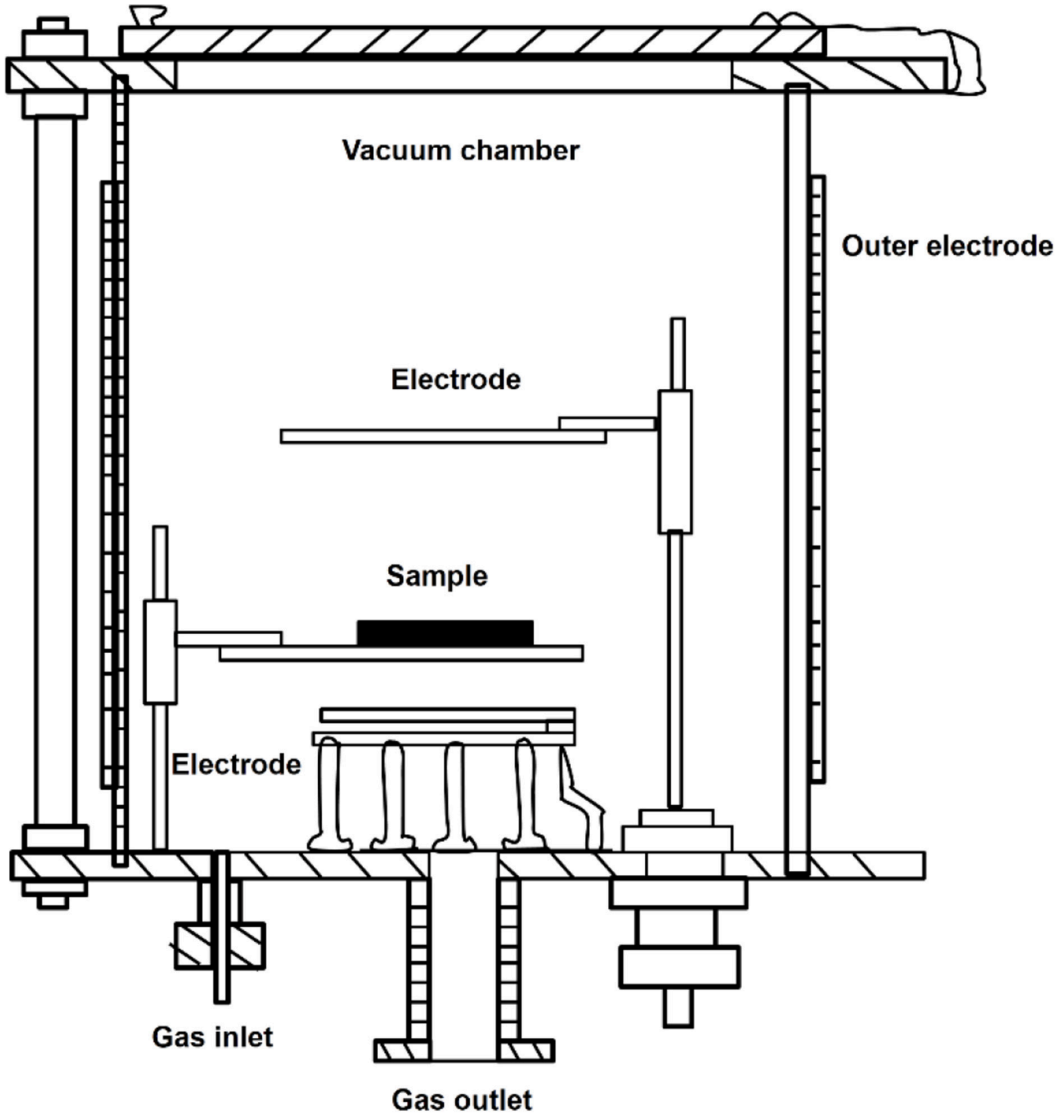
Scheme of plasma treatment device.

Plasma treatment is a straight-forward and widely used method for modifying the surface of materials to improve cell affinity of cell scaffolds (Yang et al., 2002b; Oehr, 2003). Plasma is complex system composed of neutral or excited states of atoms, molecules, free radical, electronics, ions, and radiant photon with high energy and high reactivity. It belongs to the fourth state, which is beyond the state of the solid, liquid and gas. Generally, the electromagnetic radiation, especially in ultraviolet and vacuum ultraviolet regions, is rich of plasma. By applying the plasma generator in Figure 8, plasma can be obtained using radio-frequency discharge in 0.1 ~ 100 Pa. Since surface parts of the materials are exposed to energies higher than the characteristic bonding energy of polymers, these parts undergo scission reactions and form new bonding configurations on the surface (Oehr, 2003). Functional groups such as –NH_2_, -OH, and -COOH can be grafted by plasma treatment with non-deposition gas such as ammonia, oxygen, hydrogen oxide, and so on (Yang et al., 2002b; Oehr, 2003). Because the temperature of particles in the generator is close to or slightly higher than the room temperature, it is also called low temperature plasma.

The process for plasma treatment is relatively simple. Firstly, the generator is filled with gas that cannot be polymerized, such as methane, ammonia gas, nitrogen, oxygen, and argon. Then the sample is put into the cavity of generator; by using electrostatic field, plasma particles are generated, inducing molecular excitation, ionization, and chemical bond fracture on the surface. This method sculptures the surface in the range from dozens to thousands of Egypt to form new topology structure and will not cause the thermal decomposition or ablation of the material (Oehr, 2003).

The effect of plasma modification could be optimized by changing the gas, processing time, pressure and processing power. Different gas and pressure will result in the formation of functional groups with various types and properties. The plasma processing time and power lead to different processing depth, topology structure and densities of the formed functional groups (Favia and d'Agostino, 1998; Wang et al., 2004; Wang M. J. et al., 2005).

**Figure 9:**
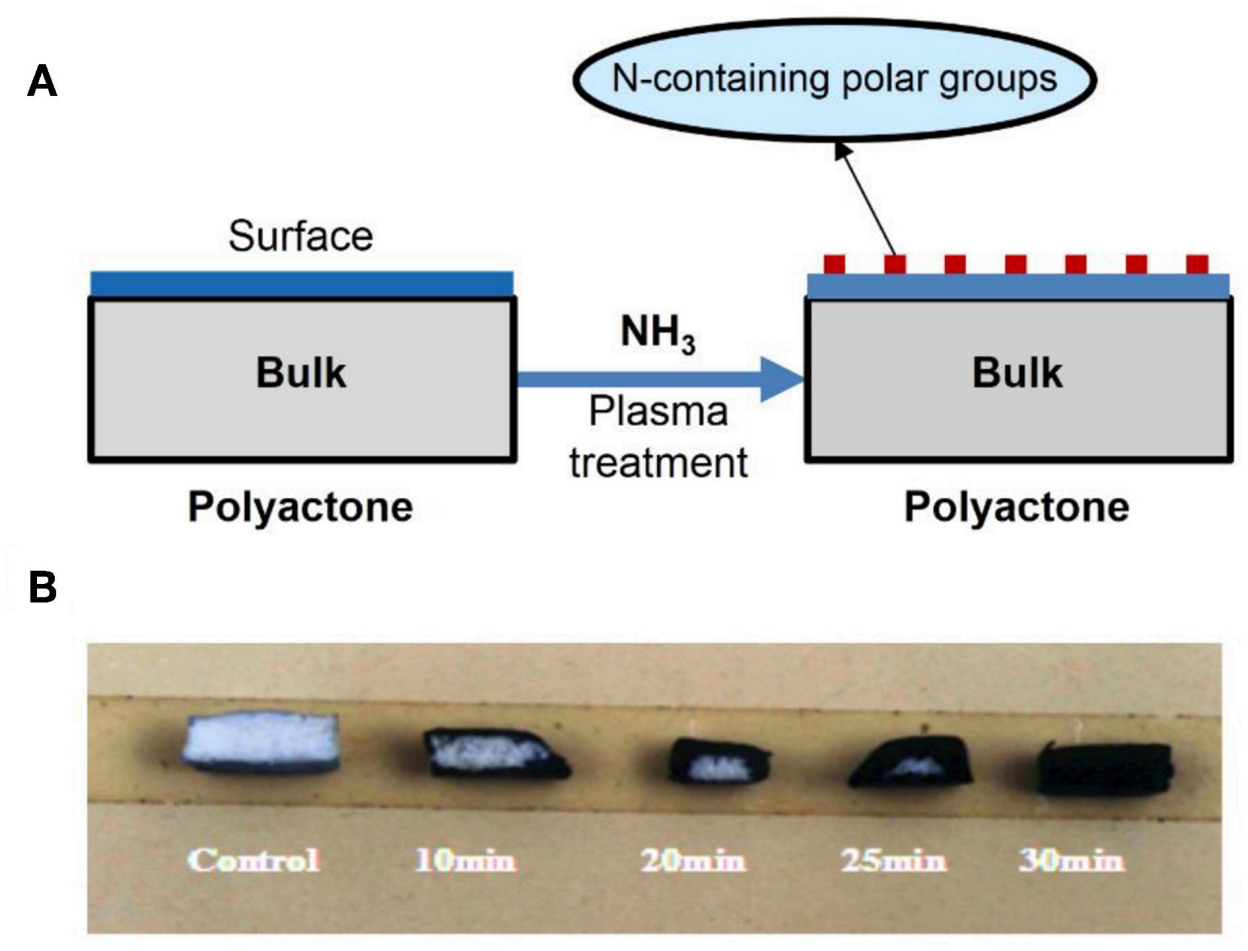
**(A)** Scheme of formation of N-containing polar groups on surface of polylactone-type polymer using ammonia plasma treatment; **(B)** the influence of plasma-treating time under power 20 W and 30 Pa of NH_3_ atmosphere on treated depth of PLGA scaffold.

**Figure 10:**
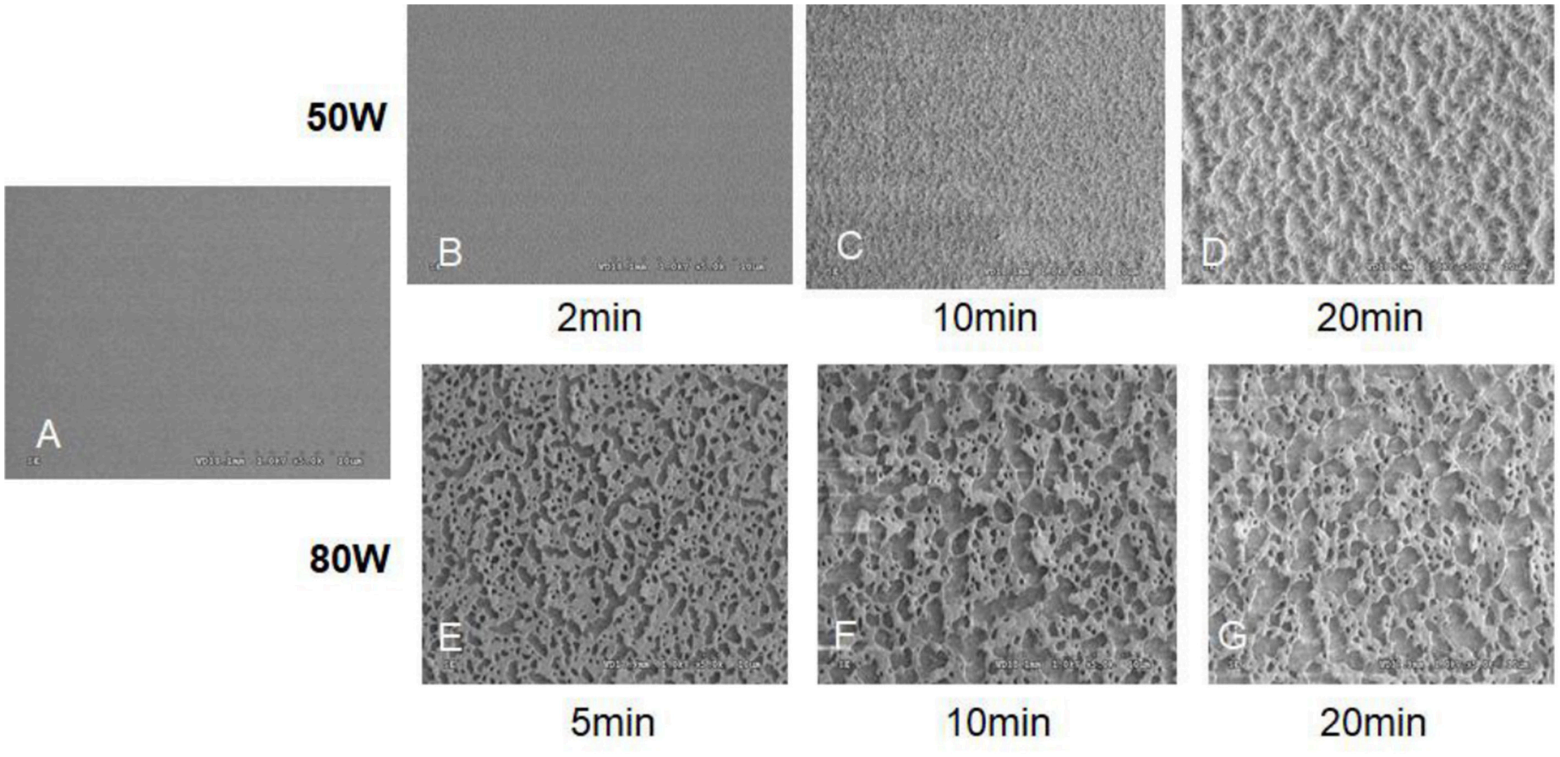
The SEM images of control and oxygen-plasma treated PLGA surface: **(A)** Control; **(B)** 50 W, 2 min; **(C)** 50 W, 10 min; **(D)** 50 W, 20 min; **(E)** 80 W, 5 min; **(F)** 80 W, 10 min; **(G)** 80 W, 20 min (Wang et al., 2004) [Readapted with permission from Wang et al. (2004). Copyright 2003 Elsevier Ltd.].

Under an ammonia atmosphere, some nitrogen-contained polar functional groups could be formed on the surface of polyester (Figure 9A). The depth of modification (Figure 9B) and the surface topology changed (Figure 10) with the increase of the plasma treatment time (Wang et al., 2004).

Compared with other methods, low temperature plasma is not only easier to operate but also more efficient in changing the hydrophilicity and electric properties. The method can avoid changing physical and chemical properties as well as morphology structure. It is also a green method without risk of pollution.

**Figure 11:**
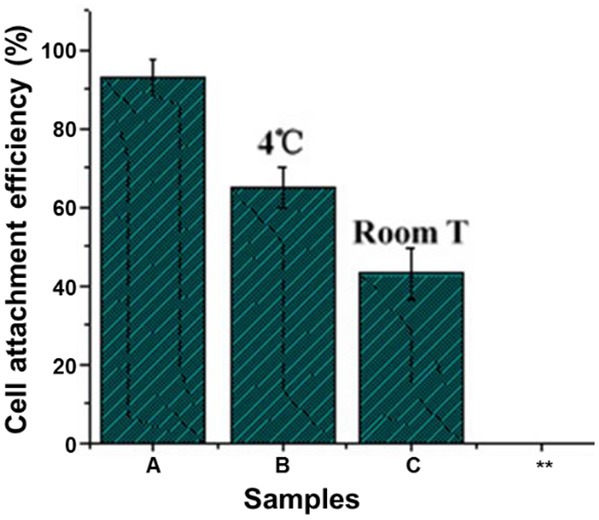
Effect of storing temperature on adherence of mice 3T3 fibroblasts on NH_3_ plasma-treated PLGA films under 20.3 N/m^2^ of shear stress for 60 min (Wang et al., 2004) (Readapted with permission from Wang et al. (2004). Copyright 2003 Elsevier Ltd.).

However, since the plasma easily moves due to the thermal action under the common temperature, the plasma on the surface would gradually migrates inside of the materials, resulting in the decreasing of functional groups on the surface and reducing attachment efficiency of the cell (Figure 11). To solve the problem, it is necessary to decrease storage time of the plasma treating product, and/or to keep the plasma treating product at low temperature (Wang et al., 2004).

#### Plasma Treatment-co-Biomolecule Anchoring Method

By using plasma treatment, functional groups with special physical and chemical properties can be generated, grafting other molecules or bioactive molecules. After the functional groups are bonded with other molecules and/or the large groups, the thermal moving of plasma will become difficult due to the increased volume, increasing the stability of the plasma treatment.

Yang et al. used nitrogen containing groups (C-N^+^, -NH-) which generated under ammonia atmosphere to fix collagen. After fixing the collagen, the plasma treatment was prolonged and the biocompatibility was improved (Yang et al., 2002a,b, 2003, 2004).

Wang et al. applied oxygen plasma to modify PLGA. They found that the surface roughness was improved after modification. What's more, the formed oxygen containing functional groups on the surface could adsorb bovine serum albumin during the cell culture process and mediate cell to adhere and grow on PLGA (Wang et al., 2004).

**Figure 12:**
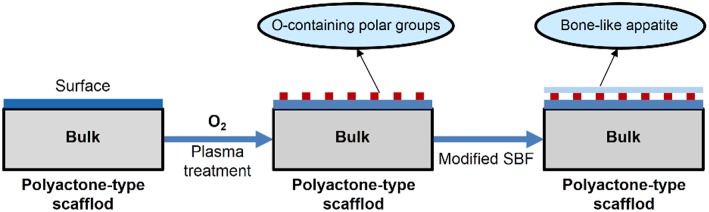
Modification of PLGA by oxygen-plasma treatment then bone-like apatite incubation in modified SBF.

As shown in Figure 12, after PLGA was performed with plasma treatment under oxygen atmosphere, a bovine serum albumin layer can be formed on the surface of PLGA. The resulting scaffold showed enhanced cell affinity with OCT-1 cells (Qu et al., 2007).

Growth factors are bio-active molecules that can influence cell growth and other functions. Growth factor was found in platelet, adult and embryonic tissues as well as many cells which differ according to types of cells. It is also one of the three main elements for Tissue Engineering. However, growth factors have poor thermal stability and easily lose biological activity at room temperature or in water (Langer and Vacanti, 1993). To keep the biological activity of the growth factor, Shen et al. fixed alkaline fibroblast growth factor (b-FGF) onto surface of the PLGA by using the plasma treatment-growth factor anchorage technique under CO_2_ (Shen et al., 2008). Then they fixed rhBMP-2 onto surface of the PLGA by plasma treatment under oxygen atmosphere (Shen et al., 2009, 2010). This surface treatment technique can not only prevent the plasma from migrating inside of the scaffold, but also keep biological activity of the fixed growth factor and achieve slow release of the growth factor (Shen et al., 2007).

Plasma has good penetrability. The plasma treatment is not limited to the cell scaffolds with smooth and/or rough surface but also can be used on materials with hollows and/or porous structure. However, it must be noted that when using the plasma treatment, the special plasma generator and gas are necessary, and control equipment is also expensive. Besides, the size of the treated material is restricted by the size of the equipment chamber.

## Conclusion

To improve biocompatibility of aliphatic polyesters, copolymerization, surface coating and grafting as well as plasma treatment can be used for the surface modification of the aliphatic polyester to optimize the properties. Chemical modification can achieve long and stable effects but is limited by the co-polymerization materials and the functional groups. Physical coating method is simple and effective, but the bonding is relatively weak, especially in water. Plasma treatment is a convenient and widely used method, but the size of the treated material is restricted. The selection of modification methods should be based on biomedical application and request.

## Author Contributions

YB did the literature search and paper writing. JM did literature research, data analysis and helped revise the paper. JB provided suggestion on biocompatibility. SW was responsible for the whole paper design and manuscript organization.

### Conflict of Interest Statement

The authors declare that the research was conducted in the absence of any commercial or financial relationships that could be construed as a potential conflict of interest.
